# TGF-β Superfamily Gene Expression and Induction of the Runx1 Transcription Factor in Adult Neurogenic Regions after Brain Injury

**DOI:** 10.1371/journal.pone.0059250

**Published:** 2013-03-21

**Authors:** Trevor T. Logan, Sonia Villapol, Aviva J. Symes

**Affiliations:** 1 Department of Pharmacology, Uniformed Services University of the Health Sciences, Bethesda, Maryland, United States of America; 2 Center for Neuroscience and Regenerative Medicine, Uniformed Services University of the Health Sciences, Bethesda, Maryland, United States of America; University of Nebraska Medical Center, United States of America

## Abstract

Traumatic brain injury (TBI) increases neurogenesis in the forebrain subventricular zone (SVZ) and the hippocampal dentate gyrus (DG). Transforming growth factor-β (TGF-β) superfamily cytokines are important regulators of adult neurogenesis, but their involvement in the regulation of this process after brain injury is unclear. We subjected adult mice to controlled cortical impact (CCI) injury, and isolated RNA from the SVZ and DG at different post-injury time points. qPCR array analysis showed that cortical injury caused significant alterations in the mRNA expression of components and targets of the TGF-β, BMP, and activin signaling pathways in the SVZ and DG after injury, suggesting that these pathways could regulate post-injury neurogenesis. In both neurogenic regions, the injury also induced expression of Runt-related transcription factor-1 (Runx1), which can interact with intracellular TGF-β Smad signaling pathways. CCI injury strongly induced Runx1 expression in activated and proliferating microglial cells throughout the neurogenic regions. Runx1 protein was also expressed in a subset of Nestin- and GFAP-expressing putative neural stem or progenitor cells in the DG and SVZ after injury. In the DG only, these Runx1+ progenitors proliferated. Our data suggest potential roles for Runx1 in the processes of microglial cell activation and proliferation and in neural stem cell proliferation after TBI.

## Introduction

Adult traumatic brain injury (TBI) is a prevalent injury that often results in permanent loss of neurological function. In cases of severe TBI, clinical treatment focuses primarily on stabilizing the patients, performing intubation and ventilation if necessary, and monitoring and managing intracranial pressure, blood pressure, oxygenation, and glycemic levels [1]. Secondary to stabilization, specific symptoms such as seizures are treated [2], but currently there are no standard clinical avenues available to facilitate repair, regeneration, or to enhance neuronal survival [1], [3]. In the days following cortical TBI, massive amounts of cell death occur in the lesion core, pericontusional region, and in distal regions, such as the hippocampus [4]. Treatments which help regenerate neurons could be beneficial, and significant recent research has focused on the possibility that the endogenous neural stem cell (NSC) population could be harnessed to stimulate regeneration and recovery of the central nervous system (CNS) following injury [5], [6]. Widespread inflammation occurs concomitantly with cell death after injury, with microglia and astrocytes becoming activated and blood borne immune cells entering the lesion. This post-injury inflammation has broad impact on processes in both the lesion area and the neurogenic regions [7]–[9].

In the adult mammalian brain, NSCs and/or neural progenitor cells (NPCs) are maintained in two neurogenic niches: the forebrain subventricular zone (SVZ) around the lateral ventricles, and the subgranular zone (SGZ) of the dentate gyrus (DG) [10], [11]. TBI increases the rates of NSC proliferation and neurogenesis in the adult mammalian SVZ and DG [12]–[14]. This injury-induced neurogenesis may contribute to the limited spontaneous recovery and post-injury maintenance of cognitive abilities seen in rodents [15], as well as to the repopulation of neurons in damaged areas [13], [16], [17]. Indeed, treatments that increase endogenous neurogenesis have also improved post-TBI recovery in adult rodents [18]–[20]. Thus, post-TBI neurogenesis represents a potential avenue for endogenous repair of tissue and recovery of cognitive functions following injury. Defining how the normal regulatory pathways of adult neurogenesis are altered by TBI is an essential step in attempting to manipulate post-TBI neurogenesis for therapeutic benefit.

Members of the transforming growth factor-β (TGF-β) superfamily of cytokines, including the bone morphogenetic proteins (BMPs), activins, and TGF-βs regulate many processes after TBI, including cell survival, gliosis, inflammation, and cell proliferation [21]–[24]. These cytokines also regulate adult NSC division and neurogenesis in uninjured animals [25]–[28], although the involvement of TGF-β superfamily members in regulating post-TBI neurogenesis has not been demonstrated. Basal BMP signaling inhibits adult NSC proliferation and keeps the majority of adult primary NSCs in a slowly dividing, quiescent state [27]. TGF-β1, 2, and 3 proteins inhibit NSC division and favor neuronal differentiation of NSCs in uninjured animals, but can increase NSC division rates in different injury contexts [28]–[31]. Activin-A is a crucial survival factor for immature neurons in the DG [32]. Most importantly, experimentally increasing or decreasing the levels of TGF-β, BMP, or Activin signaling in the neurogenic regions can have drastic effects on adult NSC division and neurogenesis [27], [28], [32]. Therefore, we investigated how CCI injury alters expression of these cytokines and their related signaling molecules in the neurogenic regions.

Runt-related transcription factor-1 (Runx1 or AML1) is a transcription factor that plays important roles in hematopoiesis [33], [34], olfactory neurogenesis [35], and neuronal development [36], [37]. Runx1 physically interacts with the intracellular Smad transcription factors (the canonical intracellular transducers of TGF-β, BMP, and activin signaling) [38], although not exclusively, and can act as a transcriptional cofactor to either promote or repress the expression of TGF-β superfamily target genes [39], [40]. Developmentally, in the mouse CNS and in the dorsal root ganglia, Runx1 is expressed in certain populations of postmitotic motor and sensory neurons, where it functions to stimulate and maintain their differentiation into particular neuronal subclasses [37], [41], [42]. It is also an important proliferative factor for Mash1-expressing neuronal progenitor cells in the embryonic olfactory bulb [35], and is expressed in adult NSC neurosphere cultures [43]. Interestingly, adult microglia derive from Runx1-expressing precursors, and Runx1 regulates microglial proliferation during development [44], [45]. Despite the fact that multiple populations of proliferative progenitor cells express Runx1, its expression has not previously been documented in the neurogenic regions of the adult brain.

In this paper we show that TBI causes major changes in the mRNA expression of many components of the TGF-β, BMP, and activin signaling pathways in both the SVZ and DG. We find that Runx1 is a novel injury-induced transcription factor expressed in NSCs of the adult SVZ and DG and is also expressed in proliferative and activated microglia in the neurogenic regions after TBI.

## Materials and Methods

### Animals and Controlled Cortical Impact (CCI) Injury

All animal studies were approved by the USUHS Institutional Animal Care and Use Committee and were conducted in accordance with the NRC guide to the Care and Use of Laboratory Animals. Eight week old male C57BL/6 mice (NCI), weighing 22–28 g, were kept under 12∶12 light and dark cycle with access to food and water *ad libitum*. Surgery was done after one week of recovery from transportation-related stress. Mice were anaesthetized with isoflurane (3% induction: 1.5% maintained) and affixed in a stereotaxic frame. Body temperature was kept constant using an isothermal heating pad (Stoelting). A craniotomy was performed above the left parietal cortex, and a mild-moderate controlled cortical impact (CCI) injury was performed centered over the somatosensory cortex (coordinates; 2 mm lateral, −2 mm posterior to Bregma) to an impact depth of 1 mm, with a 2 mm diameter round impact tip (speed 3.6 m/s, dwell time 100 msec) and angle of 12° from dura, using an electromagnetically driven CCI injury device (Impact One™ stereotaxic impactor CCI, Leica Microsystems Gmbh, Wetzlar, Germany). The removed bone was replaced but not sealed and the skin sutured. The mice were allowed to recover fully from anesthesia before transfer back to their cages. The control group for all comparisons described herein was comprised of age-matched uninjured male mice.

### Tissue Isolation and RNA Purification

For mRNA analysis, animals were sacrificed by CO_2_ inhalation and the brains were immediately removed and frozen in cold isopentane on dry ice, then stored at −80°C. Serial brain sections (300 µm thick) were cut by cryostat. Tissue-punches from the sections were taken using a 0.75 mm diameter circular tissue sampling tool (Harris Uni-Core, Sigma-Aldrich) to dissect out the SVZ and the DG only from the hemisphere ipsilateral to the injury. SVZ tissue was collected around the supralateral corner of the ventricle, beginning approximately 0.98 mm anterior and ending −0.34 mm posterior to bregma [46]. DG tissue was taken from the anterior intersection of the superior and inferior blades of the DG, at approximately 1.46 mm to 2.30 mm posterior to bregma [46]. Tissue punches from 6 animals were combined into each independent sample of either SVZ or DG to obtain sufficient amounts of RNA for PCR array analysis. Dissected tissue punches were immediately placed in Trizol reagent (Invitrogen) and stored at −80°C. Total RNA was isolated using RNeasy Micro RNA purification kits and genomic DNA removed with RNase-Free DNase (Qiagen). RNA integrity was verified by Experion gel electrophoresis analysis (Bio-Rad) and only samples with a QI of 7 or greater were utilized.

### cDNA Synthesis, qPCR, and qPCR Array Analysis

300 ng of RNA was used to generate cDNA with the RT^2^ first strand synthesis kit (SA Biosciences). Quantitative-PCR reactions were then carried out using the TGF-β/BMP signaling pathway PCR array (SABiosciences, PAMM-035), RT^2^ qPCR SYBR® Green master mixes (SABiosciences) and a Bio-Rad CFX96 real time system (Bio-Rad), following the manufacturer’s instructions. Expression of 85 genes related to TGF-β superfamily extracellular and intracellular signaling, were compared between the SVZ or DG of injured and control mice. mRNA expression levels were quantified using the ΔΔCt method, with ΔCt values being normalized to the average Ct value of the five housekeeping genes: β-glucuronidase (Gusb), hypoxanthine guanine phosphoribosyl transferase (Hprt), heat shock protein 90 kDa alpha, class B member 1 (Hsp90ab1), glyceraldehyde-3-phosphate dehydrogenase (Gapdh), and β-actin on the array. We compared gene expression values from 3 independent samples of SVZ or DG tissue from control mice to 3 samples taken from the same area in injured mice at each time point. For individual qPCR analysis of Runx1, two sets of primers were used, corresponding to mRNA isoforms driven by either the P1 promoter or by both the P1 and P2 promoter. The primer sequences were as follows: Runx1 P1 forward primer: 5′TTTCGCAGAGCGGTGAAAGA3′ reverse: 5′GCACTGTGGATATGAAGGAA3′; Runx1 P1–P2 forward primer: 5′TGGCACTCTGGTCACCGTCAT3′, reverse: 5′GAAGCTCTTGCCTCTACCGC3′.

### Bromodeoxyuridine (BrdU) Treatment

5-bromo-2′-deoxyuridine (BrdU, Sigma-Aldrich) was dissolved in 0.9% NaCl at a concentration of 10 mg/ml. In order to label all the dividing cells at each post-injury time point, on the day of sacrifice mice received four i.p. injections of the mitotic marker BrdU (100 mg/kg) delivered once every 2.5 hours over a total of 7.5 hours. Animals were euthanized 30 minutes after last BrdU injection at 1, 3, 7, 14, 30, and 60 days post-injury (dpi) (n = 5/time point).

### Double and Triple Immunofluorescent Labeling

For immunohistochemical analysis, animals were sacrificed by transcardial perfusion with 4% paraformaldehyde (PFA) in phosphate buffer. Brains were removed and placed in 4% PFA overnight, then transferred to 30% sucrose solution stored at 4°C. 30 µm thick sections were cut by cryostat and stored in a solution of 30% glucose, 30% ethylene glycol, and 1% polyvinylpyrrolidone in 0.01 M phosphate buffer at −80°C until use. Free-floating parallel sections were washed with 0.1% Triton-X100 in 0.1 M phosphate buffered saline (PBST) and blocked in PBST/10% normal goat serum (NGS) for 1 hour, then incubated overnight at 4°C in PBST/1% NGS containing Runx1 antisera (dilution 1∶2500; kind gift of Dr. Thomas Jessell,) together with one of the cell-specific marker antisera as described in Table 1. Sections were then washed in PBST three times and incubated with the corresponding secondary antibodies (Table 1) for 2 hours at room temperature. Sections were rinsed once with PBST followed by a distilled water rinse before coverslipping with ProLong Gold antifade reagent (Invitrogen). Negative control slides were blocked and then incubated only in secondary antibody solution. No cell labeling was observed in negative controls.

**Table 1:** Primary (1°) and secondary (2°) antibodies used.

1° Antibodies	Species	Dilution	Source	Reference	Cell type labeled
Runx1	Rabbit	1∶2500	Dr. Jessell	Chen 2006	Runx1+ cells
BrdU	Rat	1∶200	Accurate	OBT0030	Proliferative cells
Nestin	Mouse	1∶200	Millipore	MAB353	NSCs, intermediate progenitors
NeuN	Mouse	1∶200	Millipore	MAB377	Mature neurons
NF200	Mouse	1∶2000	Sigma	N0142	Neuronal filaments
GFAP	Mouse	1∶1000	Millipore	MAB360	NSCs, astrocytes
Sox2	Mouse	1∶500	R&D	MAB2018	NSCs, intermediate progenitors
DCX	Mouse	1∶500	Millipore	AB2253	Immature neurons
Iba1	Rabbit	1∶750	Wako	019-19741	Microglia
Mash1	Mouse	1∶300	R&D	A21244	Intermediate progenitors
S100β	Mouse	1∶500	Sigma	S2532	Mature astrocytes
**2° Antibodies**	**Species**	**Dilution**	**Source**	**Reference**	**Conjugate**
Rat IgG	Goat	1∶2000	Jack. Immuno.	112-485-167	Dylight 488
Mouse IgG	Goat	1∶1000	Invitrogen	A21121	Alexa Fluor 488
Rabbit IgG	Goat	1∶2000	Invitrogen	A11036	Alexa Fluor 568
Mouse IgG	Goat	1∶2000	Invitrogen	A11031	Alexa Fluor 568
Mouse IgG	Goat	1∶1000	Invitrogen	A21236	Alexa Fluor 647
Rabbit IgG	Goat	1∶500	Invitrogen	A21244	Alexa Fluor 647

### Quantitative Analysis of Runx1 and Iba1 Positive Cells

Quantifications of Runx1, Runx1+/BrdU+ double and Runx1+/BrdU+/Iba1+ triple-labeled cells were performed on a series of 5 randomly selected, parallel, coronal brain sections (30 µm thick), along the ipsilateral SVZ (approx. 0.86 mm rostral to −0.34 mm caudal from bregma) or DG (−1.46 mm to −2.30 mm from bregma), using a stereological approach. The first rostral SVZ section was randomly selected for staining and analysis at approximately +0.86 mm from bregma, and each subsequent section selected was 210 µm caudal to the previous section. From the DG, the first rostral section was randomly selected at approximately −1.46 mm from bregma and each subsequent section was 120 µm caudal to the previous. Five separate animals were analyzed at each timepoint after injury and for the control group. Images (20x) were acquired using an Olympus BX61 fluorescence microscope attached to a Retiga EXi Aqua CCD camera (Qimaging), using iVision software (BioVision Technologies). For the SVZ, images were centered over the supralateral corner of the SVZ. For the DG, the intersection of the superior and inferior blades of the DG was positioned at the medial edge of the visual field. Cells counts were quantified by a blinded investigator using Image J software (NIH). Total Runx1 positive cells and total Iba1-positive cells, as well as Runx1-positive cells that were double or triple labeled with Iba1 and/or BrdU were counted in each image. Quantifications of Runx1 colocalization with other cell markers (listed in Table 1) were carried out on 3–5 randomly selected coronal sections from both the SVZ and DG, from 5 control animals and 5 animals from each post-injury group. Data were expressed as the average number of positive cells per square millimeter or percentage out of total Iba1 or Runx1-positive cells.

For colocalization studies, double and triple-stained cells were analyzed using a Zeiss confocal-laser scanning microscope (LSM 510) equipped with argon and He/Ne laser emitting at 488 nm and 568 nm. Images were taken at 20×, 40× and 63× magnification. Sequential Z sections were taken and overlay projections were acquired using Leica software and cropped and adjusted using Adobe Photoshop CS5.

### Analysis of Iba1+ Cell Size and Nuclear Runx1 Staining Intensity

Iba1+ immunoreactivity was thresholded and the average area of Iba1+ cell bodies was determined in each field (20×) using iVision software. The mean intensity of nuclear Runx1 fluorescence intensity in these same Iba1+ cells was also quantified by the iVision software. Data were expressed in gray scale values. All software settings (e.g., exposure time, area etc) were identical for all brain sections and animals.

### Statistical Analysis

Quantitative data for all figures and tables are expressed as mean ± s.e.m. All statistics were performed using Prism software (Graphpad). Statistical comparison of the qPCR array datasets were performed with a one-way, two-tailed ANOVA for each individual gene, comparing the expression in control animals as well as in each post-injury group, followed by Dunnet’s post-hoc t-tests comparing each post-injury group to the control group. One-tailed student’s t-tests were used to compare the expression of Runx1 between control and 1 dpi tissue in the DG and SVZ by individual qPCR for each set of primers. One-way, two-tailed ANOVA with subsequent Dunnet’s post-hoc t- tests comparing post-injury counts to the control group were used to analyze changes in total Runx1+ and Runx1+/Iba1+ cell counts in the SVZ and DG, as well as to analyze changes in Iba1+ cell area. The mean area of Iba1+ cell bodies was correlated with the nuclear intensity of Runx1 staining using Pearson’s correlation with a two-tailed P test.

## Results

### Traumatic Brain Injury Alters mRNA Expression of Components of the TGF-β, BMP, and Activin Signaling Pathways in the Adult Neurogenic Regions

**Figure 1:**
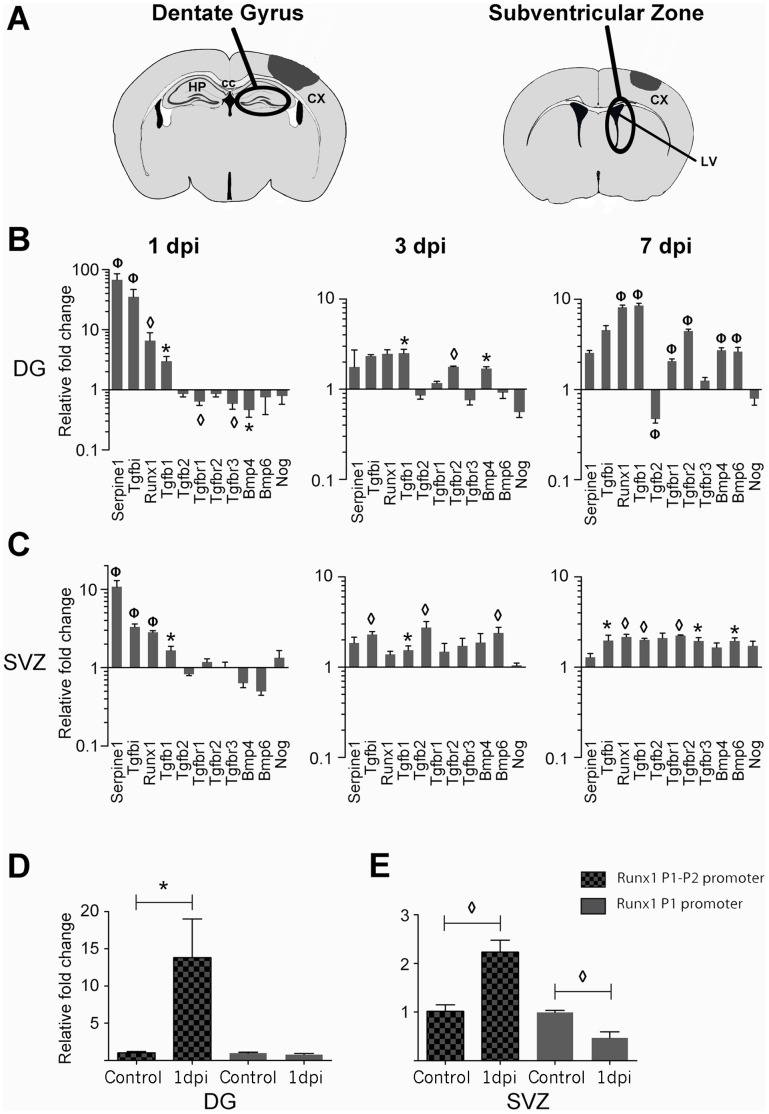
Cortical brain injury increases mRNA expression of TGF-β and BMP related molecules in the adult neurogenic areas. (A) Brain diagrams show the approximate area of the lesion penumbra at −1.82 mm from bregma for DG and +0.26 mm for SVZ, (adapted from Paxinos & Franklin, 2007). (B, C) QPCR analysis with TGF-β related PCR arrays indicates changes in RNA expression of specific genes at different times after CCI injury in either the DG (B) or the SVZ (C). Graphs (log_10_ scale) show genes whose expression was significantly altered by injury (n = 3± s.e.m.). (D, E) Runx1 mRNA expression is significantly increased at 1 and 7 dpi in both regions and is specifically upregulated in the DG (D) and SVZ (E) at 1 dpi when measured by P1–P2 primers, but not by P1 primers (n = 3± s.e.m.). Gene expression is displayed as the relative fold change in mRNA levels as compared to the level in control animals. *p<0.05, ◊ p<0.01, Φ p<0.001. Abbreviations: dentate gyrus (DG), subventricular zone (SVZ), cortex (CX), corpus callosum (CC), hippocampus (HP), lateral ventricle (LV), and days post-injury (dpi).

CCI caused significant changes in gene expression in tissue samples from the DG and SVZ, and altered mRNA expression of components of the TGF-β, activin, and BMP signaling pathways at all post-injury time points (Figure 1 and Table S1). Overall, CCI injury led to a more pronounced alteration in gene expression in the DG than in the SVZ (Figure 1). Gene expression after injury reflected a general increase in TGF-β and BMP signaling. Expression of the cytokine TGF-β1 was increased in the DG and the SVZ at 1, 3, and 7 dpi (Figure 1). TGF-β2 expression was more variable, increasing in the SVZ at 3 dpi but decreasing in the DG at 7 dpi. TGF-β receptor expression was also generally increased by injury. TβRI (Tgfbr1) and TβRII (tgfbr2) were increased by 7 dpi in the DG. TβRII (tgfbr2) and TβR3 (tgfbr3) were increased in the SVZ at 7 dpi. Rapid stimulation of TGF-β signaling after injury was shown by the large increases in TGF-β1 inducible genes at 1 dpi. Serpine1, Tgfbi, and Runx1 were potently increased at 1 dpi in the DG and SVZ. At 3 and 7 dpi Tgfbi induction persisted in the SVZ. Runx1 induction was biphasic, diminishing at 3 dpi but returning to strong induction again at 7 dpi in both the SVZ and DG. Overall, these results indicate that traumatic injury increases TGF-β signaling in neurogenic regions.

BMP signaling was also increased in the neurogenic regions after injury. At both 3 and 7 dpi, BMP4 was increased in the DG and BMP6 was increased in the SVZ (Figure 1). Several other BMPs were increased after injury (shown in Table S1), while there was no significant change in expression of the BMP inhibitor Noggin (Figure 1).

### CCI Specifically Induces Expression of Runx1 Isoforms Driven by the Proximal P2 Promoter

We were interested in proteins that could influence the injury-induced neurogenesis that occurs in both the DG and SVZ. We therefore focused on the expression of Runx1, a transcription factor that is known to be important for proliferation of hematopoietic stem cells and is expressed in mitotic neuronal precursors in development [33], [35]. To verify the qPCR array data, we performed individual qPCR on independent samples to compare the expression of Runx1 mRNA between control tissue and tissue from animals at 1 dpi. Transcription of the Runx1 gene is regulated by two alternative promoters, termed P1 (distal) and P2 (proximal). These promoters produce transcripts with different 5′UTRs and slightly different N-terminal coding sequences [47]. To determine which promoter is active after injury, we generated two sets of primers: P1 and P1/P2. The P1 primer set amplifies a region of Runx1 spanning exons 1 and 2, which are only present in Runx1 isoforms driven by the P1 distal promoter. The P1/P2 primer set amplifies a region spanning exons 4 and 5, which are present in Runx1 isoforms driven by both promoters. The P1/P2 primers amplified transcripts were significantly increased in both the DG and SVZ at 1 dpi (Figure 1D and E), whereas P1 amplified transcripts were unchanged in the DG and significantly decreased in the SVZ at 1 dpi. Therefore, our data indicate that CCI injury induces a specific increase in P2 promoter activity, driving an increase in Runx1 expression in the neurogenic regions.

### Runx1 Protein is Upregulated in the SVZ and DG after CCI Injury

**Figure 2:**
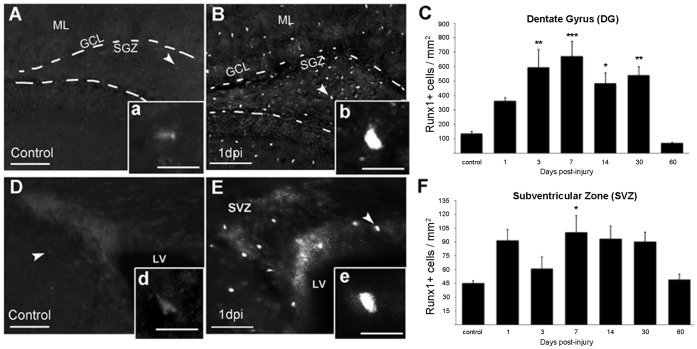
Runx1 expression in the two neurogenic areas of the adult brain. Runx1 is present at low levels in control animals in the DG (A, inset a) and SVZ (D, inset d). Runx1 expression increases in both regions after injury (DG; B, inset b and SVZ; E, inset e). (C, F) The number of cells expressing Runx1 is significantly increased in the DG at 3, 7, 14, and 30 days after injury and significantly increased in the SVZ at 7 days after injury compared with control animals. The peak of Runx1 expression in both regions was seen at 7 dpi. *p<0.05, **p<0.01 ***p<0.001, n = 5± s.e.m. Scale bars for A, B, D, E = 50 µm; for a, b, d, e = 20 µm. Abbreviations: subventricular zone (SVZ), dentate gyrus (DG), molecular layer (ML), granule cell layer (GCL), subgranular zone (SGZ), lateral ventricle (LV), and days post-injury (dpi).

To determine the spatial and temporal expression pattern of Runx1 protein, we stained brain sections taken from control mice or mice that were sacrificed at different time points after CCI injury. Cells with low-level Runx1 immunoreactivity were sparsely dispersed in regions including the DG (135±16 cells/mm^2^) and SVZ (45±3 cells/mm^2^) in control mice (Figure 2A, D). After injury, Runx1+ cells were prevalent throughout the ipsilateral hemisphere, in the SVZ and DG (Figure 2B, E), as well as the corpus callosum and prominently in the cortex surrounding the lesion (not shown). Quantitative evaluation showed that the total number of Runx1 expressing cells began increasing at 1 dpi in the DG, and statistically significant increases occurred at 3, 7, 14, and 30 dpi as compared to control mice (Figure 2C). In the SVZ, Runx1+ cell counts began increasing at 1 dpi, were significantly elevated at 7 dpi but returned to control levels by 60 dpi (Figure 2F).

### Runx1 is Expressed Predominantly in Microglial Cells in Neurogenic Regions of the Adult Mouse Brain

**Figure 3:**
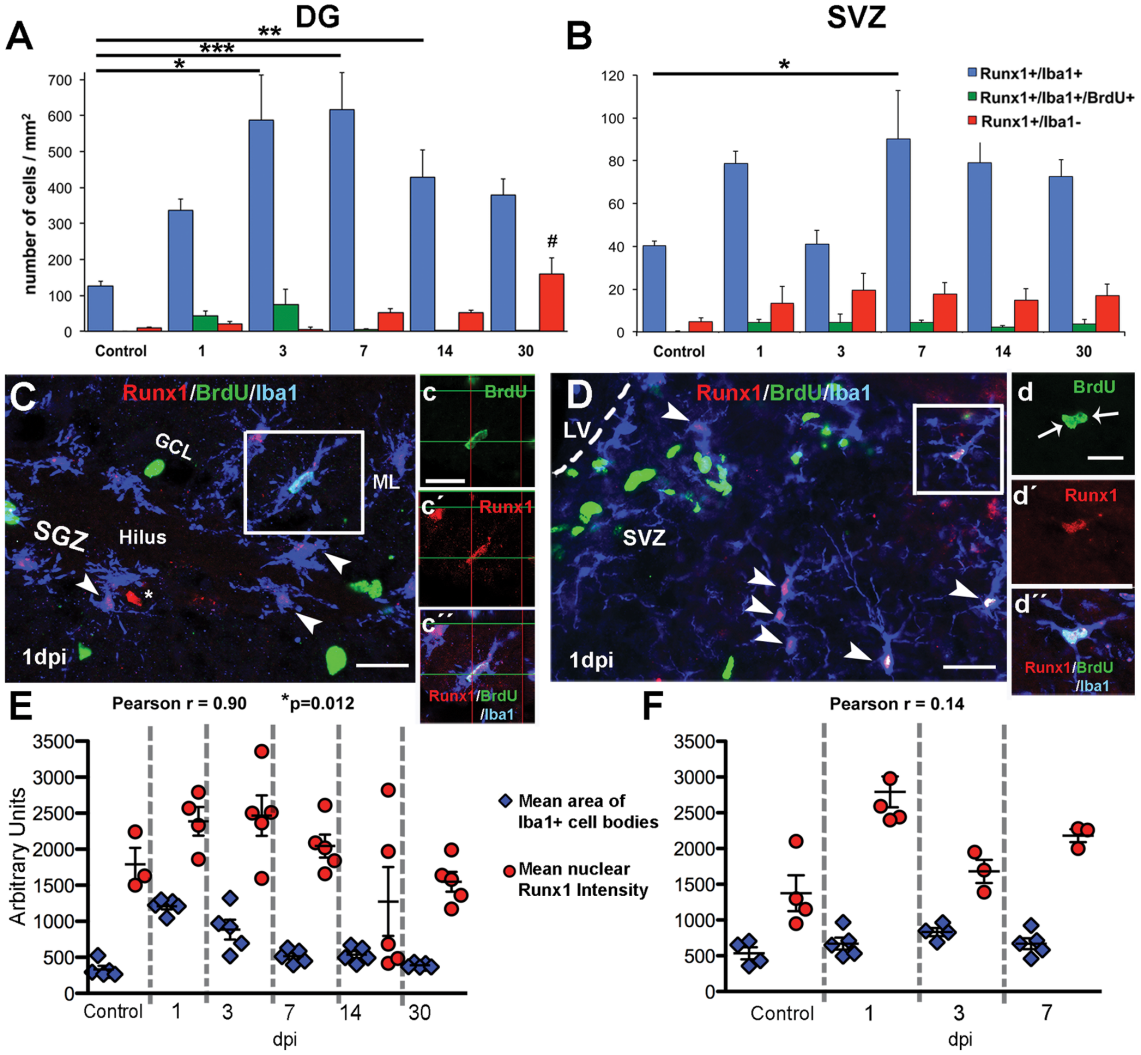
Runx1 is expressed predominantly in microglia after CCI injury in the neurogenic regions of the adult mouse. (A, B) The number of Runx1+ cells/mm^2^ identified as Iba1+ microglia (blue bars), Iba1+ microglia incorporating BrdU (green bars), or Iba1− cells (red bars) are shown in the DG and SVZ. No Iba1+ cell proliferation was observed in control animals. Asterisk indicates statistically significant difference as compared to control Runx1+/Iba1+ cell counts and number symbol indicates difference as compared to control Runx1+/Iba1− cell counts. *p<0.05, **p<0.01 ***p<0.001, and #p<0.01. n = 5± s.e.m. (C, D) Confocal micrographs of triple labeling for Runx1 (red), Iba1 (blue), and BrdU (green) positive hypertrophic microglia in the DG (C and insets c, c′, c′′) and intermediate microglia in the SVZ (D and insets d, d′, d′′) at 1 dpi. Arrowheads denote Runx1+ microglia (C, D), and an asterisk (C) denotes a non-microglial, Runx1+ cell. Scale bars for C and D = 50 µm; for c and d = 20 µm. (E, F) Graphs show the mean area of Iba1+ cell body (total pixels) and mean Runx1 immunoreactivity (pixel intensity) at different time points post-injury and in control in the DG (E) and SVZ (F). Each point represents combined data from one animal. Abbreviations: subventricular zone (SVZ), dentate gyrus (DG), granule cell layer (GCL), subgranular zone (SGZ), molecular layer (ML), lateral ventricle (LV), and days post-injury (dpi).

**Figure 4:**
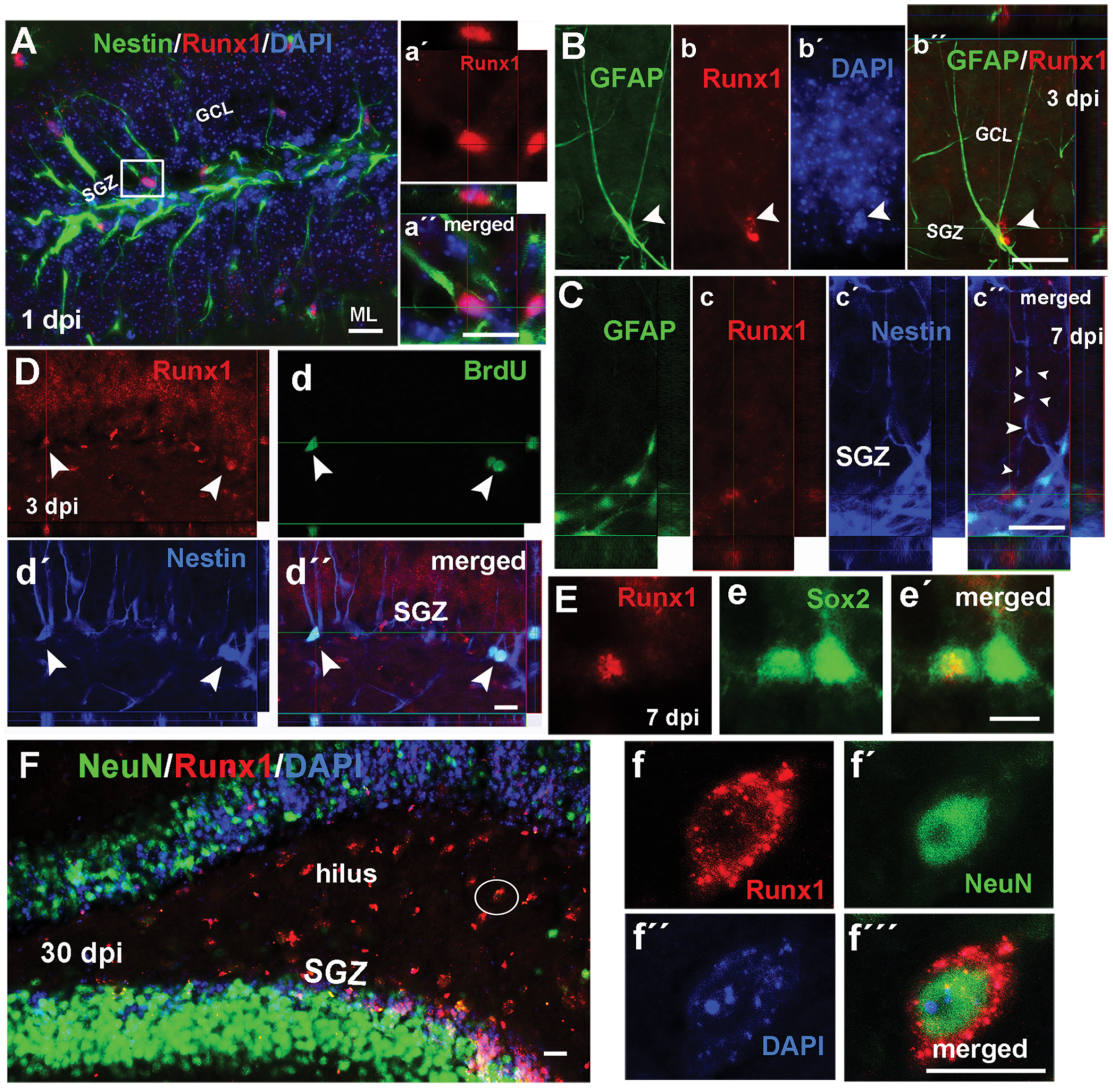
Adult hippocampal radial glia-like cells and mature neurons express Runx1. Confocal microscopy images of adult hippocampal mouse sections showing Runx1 (red) colocalized with Nestin+ (green) radial glia-like cells projecting radially from the dentate gyrus subgranular zone (SGZ) after injury (A, inset a′, a′′, DAPI in blue). These Runx1+ cells (red) often expressed GFAP (green) shown at 3 (B, b, b′, b′′, DAPI in blue) and 7 days post-injury (dpi) (C, c, c′, c′′, Nestin in blue), and extended processes through the granule cell layer (GCL) (an example is highlighted by the arrowheads in c′′). Some of these Runx1+/Nestin+ cells in the SGZ incorporated BrdU (D, d, d′, d′′). Nuclear Runx1 was also found to colocalize with Sox2 (green) expression in radial glia-like cells of the SGZ after injury (E, e, e′). At late time points (30 and 60 dpi), Runx1 was rarely expressed in the SGZ, but widely expressed in mature neurons (NeuN - green) of the dentate gyrus hilus (F, inset in f, f′, f′′, f′′′). Scale bars for A, a′′, b′′, c′′, e′, f′′′ = 20 µm; for d′′ and F = 50 µm. Abbreviations: molecular layer (ML).

After injury, the morphology of Runx1+ cells was suggestive of microglia. We therefore stained brain sections with the microglial marker, Iba1, together with Runx1. In control animals and at all post-injury time points, the majority of cells expressing Runx1 were Iba1+ microglia, in both the DG and SVZ (Figure 3A and C, and 3B and D, respectively). The total number of microglia expressing Runx1 increased following injury in a similar pattern to the number of total Runx1 expressing cells: Runx1+/Iba1+ cell counts were significantly increased over counts from control mice in the DG at 3, 7, and 14 dpi (Figure 3A) and in the SVZ at 7 dpi (Figure 3B). The percent of microglia that expressed Runx1 was significantly increased by injury, particularly at 1 dpi, increasing from ∼47% to ∼99% in the DG and from ∼11% to ∼64% in the SVZ (Table 2). There was a small number of Runx1+/Iba1- cells in both regions (Figure 3A and B) that varied over time. In the DG, the number of Runx1+/Iba1− cells was significantly increased at 30 dpi (Figure 3A). As will be discussed, Runx1 was also expressed by some neurons and neural progenitor cells of the DG after injury, and in a small percentage of astrocytes (data not shown). There was a particularly large population of Runx1+ neurons in the DG hilus region at 30 dpi, which likely comprised the majority of these Runx1+/Iba1− cells at this time point (Figure 4F). In the SVZ, these Runx1+/Iba− cells were also most likely neural progenitor cells, ependymal cells, or astrocytes.

**Table 2:** Runx1 expression in the microglial cell populations of the DG and SVZ.

		*Control*	*1 dpi*	*3 dpi*	*7 dpi*	*14 dpi*	*30 dpi*
**DG**	% Runx1+/Iba1+ out of total Iba1+ population	46.6±6%	99.0±0.6%	83.2±6%	81.5±5%	69.3±13%	80.2±7%
	% of BrdU+/Iba1+ cells expressing Runx1	n/a	100%	94%	100%	100%	100%
	Runx1+/Iba1+/BrdU+ cells/mm^2^	0±0	42.3±13.6	74.5±41.8	5.6±1.8	2.0±0.69	2.7±1.0
**SVZ**	% Runx1+/Iba1+ out of total Iba1+ population	10.6±1%	64.4±2%	28.6±5%	23.4±5%	30.2±4%	16.7±2%
	% of BrdU+/Iba1+ cells expressing Runx1	n/a	100%	92%	94%	100%	100%
	Runx1+/Iba1+/BrdU+ cells/mm^2^	0±0	4.3±1.5	4.5±3.9	4.3±1.2	2.2±0.72	3.8±1.9

The percentage of cells expressing the indicated markers is shown at different time points (n = 5± s.e.m.).

### Proliferating and Reactive Microglia Cells in Neurogenic Areas Express Runx1 after Cortical Injury

Resting microglia in uninjured adult animals have a small nucleus and cell body and multiple branched processes extending outward. In response to injury, microglia become activated and hypertrophic, their processes thicken, branch, and retract, and their cell body increases in size, causing the cells to become amoeboid in shape (for a review see [48]). The Iba1+ cells clearly exhibited these characteristics. The average size of Iba1+ cell bodies that expressed Runx1 was significantly increased in the DG at 1 and 3 dpi (p<0.01), indicating these microglia were activated. In accordance, the average intensity of Runx1 nuclear staining in Iba1+ microglia of the DG correlated significantly with changes in the average size of the microglial cell body from 1–30 dpi (Figure 3E, Pearson r = 0.90, p = 0.01). The SVZ microglia, however, were less affected by the injury: the average cell body size of SVZ microglia was unchanged after CCI (Figure 3F). Our observations indicated that microglia in the SVZ were mildly activated after injury, although their activation was mostly to an intermediate phenotype whereby their processes thickened and branched without noticeable change in the size of their cell body (data not shown). In the SVZ there was no correlation between the intensity of microglial nuclear Runx1 staining and the size of the microglial cell bodies (Figure 3F).

To determine whether Runx1 expression was associated with cell proliferation, mice were injected with BrdU (100 mg/kg) in a saturation protocol to label all dividing cells at a single time point. On the day of sacrifice at each time point, we injected mice four separate times at 2.5 hour intervals, and sacrificed them 30 minutes after the last injection. Immunohistochemical triple labeling was then performed for Runx1, BrdU, and Iba1. No microglial proliferation was observed in the DG of control mice, but after injury a large population of Runx1+/Iba1+/BrdU+ cells were detected, particularly at 1 and 3 dpi, with a small but sustained population of these cells noticeable until 30 dpi (Figure 3A, C; Table 2). Microglial proliferation was also not observed in the control SVZ, but following CCI, a small number of Runx1+/Iba1+/BrdU+ cells were detected (Figure 3B, D; Table 2). This slight increase was sustained until 30 dpi. Strikingly, in both the DG and SVZ at all time points, virtually all of the microglia that incorporated BrdU after injury expressed Runx1 (Table 2).

### Proliferative Hippocampal Neural Progenitor Cells and Neurons Express Runx1 after Injury

Examination of Runx1 staining in the SVZ and DG showed that a small number of Runx1+ cells had a different morphology from microglia, did not express Iba1, and were localized more specifically in the neurogenic niches. Therefore, to examine whether Runx1 was expressed by neural stem or progenitor cells, we performed immunohistochemical staining of the SVZ and DG with antibodies against Nestin, GFAP, Sox2, Mash1, DCX, NeuN, or S100β, together with Runx1. A subpopulation of cells expressing the neural stem and progenitor cell marker protein Nestin in the DG subgranular zone (SGZ) co-expressed Runx1 after injury (Figure 4A, C, D, Table 3). These cells bore the radial glia-like morphology of adult NSCs, with the soma in the SGZ and a radial process extending into the granule cell layer. Many of these Nestin+/Runx1+ cells co-expressed GFAP (Figure 4B, C). Runx1 was also expressed in a subpopulation of Sox2-positive radial cells in the SGZ after injury (Figure 4E). Many NSC marker proteins are also expressed in reactive astrocytes after injury. However, GFAP+/Runx1+ cells of the SGZ did not co-express the mature astrocyte marker S100β (Figure S1), indicating that they were in fact NSCs and not reactive astrocytes.

**Table 3:** Colocalization of Runx1 with cell-type markers in the neurogenic regions.

Colocalization of Runx1 with progenitor/stem cells markers								
		*Control*	*1 dpi*	*3 dpi*	*7 dpi*	*14 dpi*	*30 dpi*	*60 dpi*
**DG**	Nestin	**−**	**++**	**++**	**++**	**+/−**	**−**	**−**
	GFAP	**−**	**+**	**+**	**+**	**+/−**	**−**	**−**
	Sox2	**−**	**++**	**++**	**++**	**−**	**−**	**−**
	Mash1	**−**	**−**	**−**	**−**	**−**	**−**	**−**
	DCX	**−**	**−**	**−**	**−**	**−**	**−**	**−**
	NeuN	**−**	**−**	**−**	**+/−**	**++**	**+++**	**++**
	Nestin/BrdU	**−**	**+**	**+**	**+**	**+**	**−**	**−**
**SVZ**	Nestin	**−**	**+**	**++**	**+**	**+/−**	**+/−**	**−**
	GFAP	**−**	**+/−**	**+/−**	**−**	**−**	**−**	**−**
	Sox2	**−**	**−**	**−**	**−**	**−**	**−**	**−**
	Mash1	**−**	**−**	**−**	**−**	**−**	**−**	**−**
	DCX	**−**	**−**	**−**	**−**	**−**	**−**	**−**
	NeuN	**−**	**−**	**−**	**−**	**−**	**−**	**−**
	Nestin/BrdU	**−**	**−**	**−**	**−**	**−**	**−**	**−**

The degree of colocalization is graded as;+++(high),++(moderate),+(occasional), +/− (in rare cases), and – (non existing).

At 1, 3, and 7 dpi some of the Nestin and Runx1 co-expressing cells of the SGZ incorporated BrdU, indicating that Runx1 is expressed in a proliferative population of neural stem or progenitor cells after injury (Figure 4D; Table 2). We did not observe Runx1+ cells in the DG co-labeled with the immature neuroblast marker DCX+ or with the intermediate neural progenitor cell marker Mash1 at any time point studied (Figure S2).

In later stages, at 14, 30, and 60 dpi, Runx1 expression was colocalized with NeuN in neurons of the DG, mainly in the hippocampal hilus at 14, 30 and 60 dpi (Figure 4F, Table 3). Taken together, these findings suggest that Runx1 might regulate an early event in the response of the NSC population to CCI injury, and in addition, Runx1 might have a later role during neuronal differentiation in the hilus. A summary of Runx1 colocalization with the NSC markers in the DG and SVZ is shown in Table 3.

### Runx1 Expression is Induced in Neural Progenitor Cells of the Subventricular Zone after Injury

**Figure 5:**
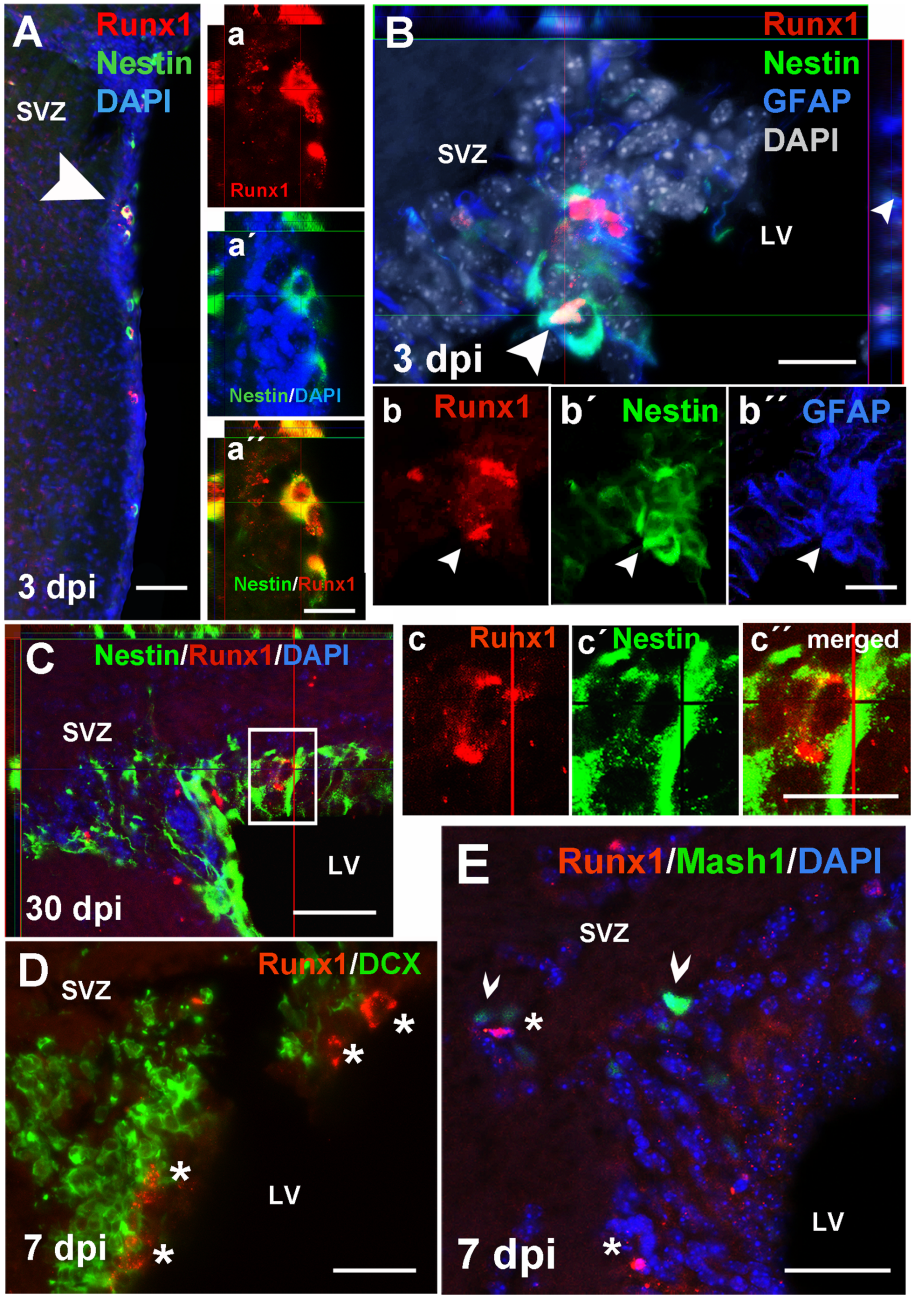
Runx1 is expressed by a subpopulation of putative neural stem cells in the subventricular zone. Runx1 (red) was co-expressed with Nestin (green) in cells of the lateral and apical subventricular zone after injury, between 3 dpi (A, inset a, a′, a′′) and 30 dpi (C, inset c, c′, c′′). Some of these cells also expressed GFAP (B, inset b, b′, b′′). Runx1 was not expressed by DCX+ (D, green) or Mash1+ (E, green) cells after injury, but was often immediately adjacent to clusters of DCX+ cells (D). Scale bars for B, a′′, b′′, c′′ = 20 µm; for A, C, D, and E = 50 µm. Abbreviations: subventricular zone (SVZ), lateral ventricle (LV), days post-injury (dpi).

Runx1 expression was observed in Nestin+ cells of the SVZ at 3, 7, 14, and 30 dpi (Figure 5A–C and Table 3). Interestingly, Runx1 expression was predominantly cytoplasmic in these SVZ cells, in contrast to its nuclear localization in the DG, and was usually observed as punctuate staining in the soma around the nucleus. Some, but not all of these Runx1+/Nestin+ double-labeled cells co-expressed GFAP (Figure 5B). Unlike Nestin+/Runx1+ cells in the DG, we did not detect BrdU incorporation in these SVZ cells at any time point. We also did not see Runx1 co-expressed with Mash1 or DCX in the SVZ, although Runx1 was often expressed in cells immediately adjacent to clusters of DCX+ cells, a characteristic of Type B NSCs [49] (Figure 5D, 5E). Our data indicate that in the SVZ, Type B neural stem or progenitor cells early in the NSC lineage express Runx1.

## Discussion

In this paper we demonstrate that CCI increases the expression of TGF-β and BMP cytokines in neurogenic regions in adult mice for up to a week after TBI. We also show that Runx1 is an early injury-induced transcription factor expressed in both microglia and NSCs in the adult neurogenic niches. Our data indicate that Runx1 expression is associated with microglial proliferation and activation, processes which themselves impact post-injury cell survival and neurogenesis. TBI also induced nuclear Runx1 expression in subpopulations of proliferative NSCs in the DG and cytoplasmic Runx1 expression in non-proliferative NSCs of the SVZ, indicating that Runx1 expression could directly regulate to post-injury neurogenesis.

TGF-β superfamily cytokines are essential regulators of adult neurogenesis, and can also mediate changes in neurogenesis in response to injury [25]–[27]. We have shown that expression of TGF-β1, TGF-β receptors, and TGF-β target genes are increased in the SVZ and DG after traumatic injury, with TGF-β responsive genes showing a dramatic induction as early as 1 dpi. The role of TGF-β in regulation of neurogenesis is complex. The majority of published work indicates that TGF- β signaling is inhibitory to adult neurogenesis, with several studies showing that chronic overexpression or infusion of TGF-β1 to uninjured rodents leads to drastically reduced hippocampal and SVZ neurogenesis [28], [29], [50]. This is mediated in part through a direct antiproliferative effect of TGF-β1 on adult NSCs, which halts their cell cycle progression in the G0/1 phase [28], [29], and can prevent the differentiation and progression of Sox2+ NSCs through the neural progenitor lineage [50]. Conversely, a few publications have indicated that TGF-β1 can increase NSC proliferation and neurogenesis when administered to animals after certain inflammatory or ischemic injuries [30], [51], [52]. Additionally, Smad3 null mice, which have defective TGF-β signaling, show reduced DG and SVZ neurogenesis, though this effect may also be due to impaired activin signaling [53]. Therefore, upregulation of TGF-β cytokines and receptors after injury has the potential to affect multiple cell types in the neurogenic niche. This injury–induced upregulation of TGF-β may have multiple opposing effects on neurogenesis. TGF-β1 could facilitate neurogenesis through its anti-inflammatory actions, while inhibiting it through its direct anti-mitotic effects on NSCs. Further, TGF-β1 has neuroprotective functions [51], [54], [55], and could enhance survival of newborn neurons.

Enhanced TGF-β signaling after CCI injury is demonstrated by the strong induction of two TGF-β target genes, Serpine1 and Tgfbi [56], [57]. Both genes are primarily expressed by astrocytes after CNS injury [56]–[58]. Serpine1 (also called PAI-1) is an extracellular serine protease inhibitor whose major targets include urokinase type plasminogen activators (uPA) and tissue-type plasminogen activators (tPA) (reviewed in [59]). Serpine1 is expressed after CNS injury, can protect neurons against excitotoxic or ischemic lesions [56], [60], and may also modulate microglial activation and migration [61], Serpine1 is therefore part of the endogenous neuroprotective response to injury. However, it has recently been suggested that Serpine1 may exacerbate vascular disruption and worsen outcome in certain ischemic injury models [60], [62]. Tgfbi is a secreted extracellular matrix (ECM) protein whose function is not completely clear. It can act as a ligand for several integrins (for a review see [63]) and can regulate the migration/cell adhesion properties of cells [64]. Thus, Tgfbi may influence the migratory response of CNS or immune cells after injury, or participate in the post-injury tissue remodeling process.

BMP signaling is a critical and highly regulated modulator of normal adult neurogenesis, and has well documented inhibitory effects on adult NSC proliferation and neurogenesis [27], [65], [66] Increased expression of the BMP inhibitor noggin mediates the elevation in neurogenesis that occurs after voluntary exercise [26], demonstrating that alterations to BMP signaling can underlie changes in neurogenesis. However, our results demonstrate increased expression of many BMP cytokines in the neurogenic regions, without any significant change in noggin levels. Post-TBI neurogenesis therefore proceeds despite increased local levels of BMP expression.

Increased NSC proliferation can begin as early as 4 hours after CCI injury, and persists at 7 dpi [13], [14]. Since both TGF-β and BMP can act directly on NSCs to impede their proliferation [27], [29], the increase in expression of these cytokines from 1 to 7 dpi suggests that the environment of the neurogenic niche after injury is not optimal to maximally promote endogenous neurogenesis. Therefore, pharmacologic manipulations to inhibit signaling by TGF-β and BMP proteins in the neurogenic regions could be beneficial to further enhance post-injury neurogenesis and recovery. However, these cytokines regulate many processes after CNS injury, and can have beneficial neurotrophic and anti-inflammatory effects [51], [67], suggesting that combinatorial treatment of TGF-β or BMP signaling inhibitors along with anti-inflammatory and/or neurotrophic treatments may be necessary.

Our qPCR array data show only global changes from the whole of the DG or SVZ, which are obviously composed of many different cell types. They may therefore not reflect smaller more localized changes specifically in microenvironment of the NSCs within the DG or SVZ. The predominant cell type in the DG, the mature neurons of the granule cell layer, may contribute more to changes in RNA expression than the neural stem cells of the SGZ, and local microglia and astrocytes would also contribute. RNA isolated from whole SVZ would reflect changes in ependymal cells, astrocytes and microglia in addition to the NSCs.

CCI strongly induced Runx1 protein expression in cells of both neurogenic regions. Runx1 interacts physically with intracellular Smad proteins and can potentiate Smad activation of target genes [39], [40]. It is therefore possible that Runx1 may affect the TGF-β/Activin or BMP Smad signaling pathways after injury. We found Runx1 expression to be a widespread, early component of the microglial response to TBI, primarily in the DG, which is near the injury site, and to a more limited degree in the more distal SVZ. Runx1 is often expressed in hematopoietic cells, but its expression in microglia has only recently been appreciated. CNS microglia derive from a Runx1 expressing precursor population [44]. Further, a recent publication showed that Runx1 regulates the proliferation and activation of early postnatal microglia in the developing CNS [68]. In this paper, Zusso and colleagues show that Runx1 inhibits proliferation and activation of early postnatal microglia as they move from an amoeboid, proliferative state to the ramified, quiescent state characteristic of resting adult microglia [68].

In control animals, we found low-level Runx1 expression in roughly 45% and 10% of the total DG and SVZ microglial populations, respectively, and these cells were predominantly ramified. CCI induced dramatic increases in microglial Runx1 expression as early as 24 hours after injury. At this time, the fraction of microglia that expressed Runx1 increased to near 100% in the DG and to 65% in the SVZ. This occurred concomitantly with an increase in the intensity of their nuclear Runx1 expression, which correlated with an increase in microglial hypertrophy in the DG. At all post-injury time points and in both neurogenic regions microglial BrdU incorporation was strongly associated with Runx1 expression, with the percentage of BrdU-incorporating microglia that expressed Runx1 remaining at or near 100% at all post-injury time points, even out to 30 dpi, when the fraction of Iba1 cells expressing Runx1 returned to control levels. The thorough, robust, and early nature of Runx1 expression in microglia, together with its high expression in proliferating microglia after injury places it appropriately to act as a driver of post-injury microglial activation in adult mice. Runx1 is a developmental regulator of microglial activation and proliferation, and has also been found to be upregulated in adult spinal cord microglia following sciatic nerve injury, supporting the idea that it is expressed as part of the injury response [68].

Yet, given that Runx1 was expressed at low levels in ramified microglia of uninjured animals, and that its post-injury expression in the SVZ did not correlate with increased size of the microglial cell bodies, Runx1 expression alone is clearly not sufficient to induce microglial activation. However, in our injury paradigm, the DG was located much closer to the actual site of impact than the SVZ. SVZ microglia were less activated after injury, having no change in the size of their cell bodies. Additionally, a smaller fraction of the SVZ microglial population expressed Runx1, with a peak of 64% expressing it at 1 dpi and only 10–30% at other time points. The increase in the total number of Iba1+/Runx1+ cells after injury was also much smaller in the SVZ than in the DG. The fact that a correlation was observed in the DG and not in the SVZ may indicate that Runx1 is part of the microglial activation pathway, and perhaps necessary for the process, but requires other signals that are present in the vicinity of the lesion.

An alternative explanation for Runx1 function in microglia is that Runx1 may be part of a feedback inhibition response to activation. In such a scenario Runx1 would be expressed after injury to inhibit microglial activation, as it does during cortical development, to prevent an excessive microgliosis response [68], and low Runx1 expression in ramified adult microglia would help maintain their resting state. In other cell types, Runx1 has been shown to either promote or inhibit proliferation [69]–[72]. Thus, a third possibility is that Runx1 is a necessary transitional component for microglia to move in either direction between a quiescent, ramified state and a proliferative, activated state. Transgenic mouse experiments with temporal microglia-specific knockouts of Runx1 after injury will be necessary to elucidate the role of Runx1 and determine whether it is necessary for the activation or suppression of microglial reactivity and proliferation in adults.

We also found that Runx1 is co-expressed with several well-characterized markers of adult NSCs after CCI injury, indicating that Runx1 may have a direct role in regulating post-injury NSC behavior. The mechanisms driving post-injury neurogenesis are largely unknown, and it is important to define novel factors involved in the process. Runx1 is expressed by neural progenitor cells of the embryonic olfactory bulb to promote their proliferation, and exogenous Runx1 expression increases proliferation of embryonic neural progenitor cells derived from either the olfactory epithelium or cerebral cortex [35]. In cell cultures of adult NSCs, high Runx1 mRNA expression is associated with cell proliferation, and Runx1 mRNA levels drop when the cells are stimulated to differentiate [43]. However, to our knowledge Runx1 expression has not been previously documented in proliferative populations of adult neural progenitor cells.

After CCI, Runx1 is expressed in subpopulations of Nestin+/GFAP+ Nestin+/GFAP-, and Sox2+ cells, specifically in the SGZ of the adult DG. These cells have the distinct radial-glial like morphology of type 1 adult NSCs [11], [73], [74], and some of these cells incorporated BrdU. Within the population of adult SGZ neural progenitor cells, GFAP expression is characteristic of type 1 quiescent adult radial NSCs, while Nestin and Sox2 are expressed in both type 1 NSCs and in the more rapidly dividing type 2 progenitor cells [11], [75]. However, Runx1 was not co-expressed with DCX or NeuN in the SGZ or granule cell layer of the DG, suggesting that Runx1 is expressed in early stages of NSCs and is switched off as they begin differentiating into immature migratory neuroblasts or neurons. It is currently unclear whether adult NSCs *in vivo* constitute a single homogenous population, or rather many different populations of more restricted NSCs and NPCs [11]. Thus, Runx1 may be expressed in a specific subpopulation of NSCs to regulate their proliferation and/or differentiation after TBI.

At later time points after injury there was a consistent population of mature NeuN-labeled neurons in the hilus adjacent to the SGZ that expressed somatic and nuclear Runx1. It is possible that Runx1 expression is repressed as NSCs differentiate into neuroblasts, and re-expressed as these neuroblasts become mature neurons in this region. Alternatively, these hilar neurons may be a population of mature neurons unrelated to the NSCs that expressed Runx1 at earlier time points.

In the SVZ, Runx1 was also expressed by Nestin+/GFAP+ and Nestin+/GFAP- cells after injury, but not by Mash1+ cells or DCX+ migratory neuroblasts. The Nestin+/Runx1+ cells were often immediately adjacent to clusters of DCX+ neuroblasts, which suggests that these are type B NSCs [49]. Therefore, cortical injury induces Runx1 expression early in the SVZ NSC lineage, and its expression is downregulated upon progression to type C, Mash1+ progenitors. In contrast to the DG, Runx1 expression in the SVZ was primarily cytosolic, and the Nestin+/Runx1+ cells did not incorporate BrdU, indicating they were not proliferative. Nuclear expression in the NSCs of the DG may promote proliferation, whereas cytoplasmic expression in the NSCs of the SVZ may serve a different purpose. Sequestration of Runx1 in the cytoplasm of myeloid and leukemic cell lines can inhibit cell proliferation [76]. Additionally, cytoplasmic Runx1 in hematopoietic stem cells reduces NF-κB (NF-κB) signaling, by inhibiting the activity of IκB-kinase [77]. As NF-κB signals may promote proliferation of NSCs [78], it is possible, that cytoplasmic Runx1 is sequestered in the cytoplasm in NSCs of the SVZ to reduce their proliferation.

The induction of neurogenesis after injury in the SVZ and DG may contribute to the ability of the brain to recover from the insult. Understanding the signaling environment of the neurogenic niche and the mechanisms by which neurogenesis is stimulated are paramount in the effort to enhance this endogenous recovery process. We show that CCI elevates potentially inhibitory TGF-β and BMP cytokine expression in the neurogenic regions, suggesting that post-injury neurogenesis could be enhanced by manipulating these signaling pathways. We also demonstrate that the transcription factor Runx1 is induced in both NSCs and microglia in the neurogenic regions after injury. Our results suggest that Runx1 is involved with microglial activation following injury, and is expressed in a proliferative population of hippocampal NSCs. In the future, cell-specific Runx1 gain- and loss-of-function studies will be extremely interesting to further clarify the role of Runx1 in adult microglial activation, post-injury neurogenesis, and the relationship between the two processes. We expect that Runx1 and its target genes could present valuable targets for therapeutic manipulations in instances of pathologic microglial activation, and in therapies designed to stimulate NSC proliferation.

## Supporting Information

Figure S1 **Reactive astrocytes and progenitor/stem cells expressing Runx1 protein in dentate gyrus of the adult mouse.** Merged image (A) showing reactive astrocytes which did not express Runx1 protein (arrows) (Runx1−/GFAP+/S100β+) and progenitor/stem cells expressing Runx1 protein (arrowheads) (Runx1+/GFAP+/S100β−) in the dentate gyrus (DG) at 1 day post-injury (dpi). S100β immunoreactivity (B, green) is present in most GFAP-expressing reactive astrocytes, but is not colocalized with Runx1 protein in the SGZ (C, red). GFAP immunoreactivity (D, blue) is detected in both S100β+ astrocytes, and with Runx1 protein in progenitor/stem cells. Scale bar = 50 µm. Abbreviations: subgranular zone (SGZ), granule cell layer (GCL).
(TIF)

Figure S2 **Runx1 is not expressed by DCX+ or Mash1+ cells of the DG.** Merged images showing Runx1+ cells (arrowheads) and DCX+ (A) or Mash1+ (B) cells (arrows) in the dentate gyrus at 1 day post-injury (dpi). Scale bars = 20 µm. Abbreviations: granule cell layer (GCL), subgranular zone (SGZ).
(TIF)

Table S1 Expression of TGF-β superfamily genes after CCI. The mRNA expression levels of all genes measured by qPCR array analysis are shown at 1, 3, and 7 dpi in the DG (A) and SVZ (B). mRNA levels are expressed as relative fold change from their expression in the respective control tissues. *p<0.05, **p<0.01, ***p<0.001.
(XLSX)
