# Supplementary figures and images for: TGF-β Superfamily Gene Expression and Induction of the Runx1 Transcription Factor in Adult Neurogenic Regions after Brain Injury
Source: PLoS One. 2013 Mar 21;8(3):e59250. doi: 10.1371/journal.pone.0059250 (PMC3605457; doi:10.1371/journal.pone.0059250)

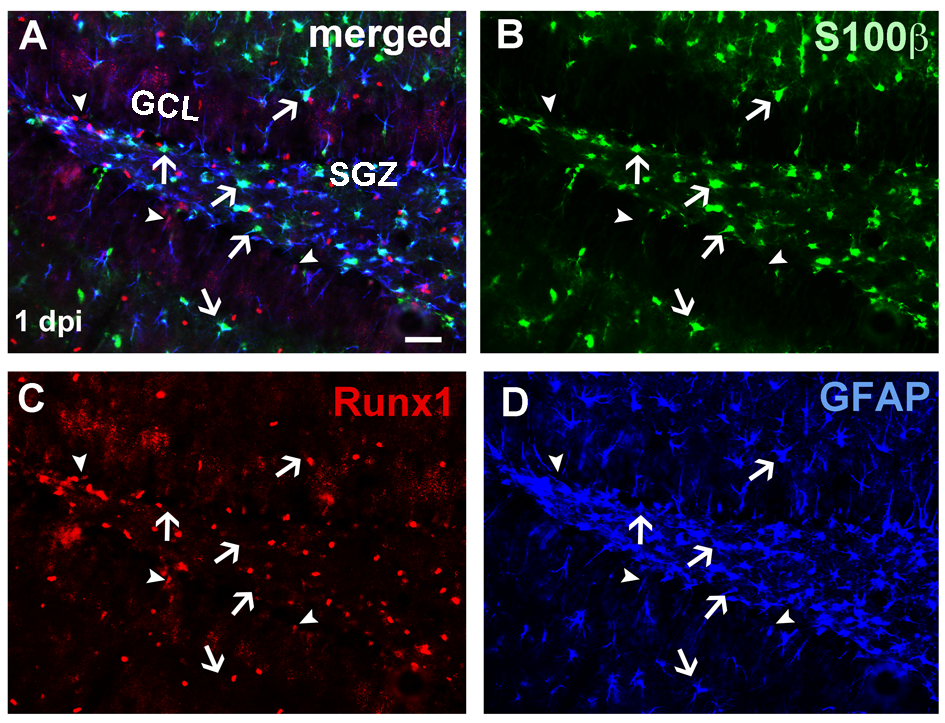

Supplement: Figure S1 — Reactive astrocytes and progenitor/stem cells expressing Runx1 protein in dentate gyrus of the adult mouse. Merged image (A) showing reactive astrocytes which did not express Runx1 protein (arrows) (Runx1−/GFAP+/S100β+) and progenitor/stem cells expressing Runx1 protein (arrowheads) (Runx1+/GFAP+/S100β−) in the dentate gyrus (DG) at 1 day post-injury (dpi). S100β immunoreactivity (B, green) is present in most GFAP-expressing reactive astrocytes, but is not colocalized with Runx1 protein in the SGZ (C, red). GFAP immunoreactivity (D, blue) is detected in both S100β+ astrocytes, and with Runx1 protein in progenitor/stem cells. Scale bar = 50 µm. Abbreviations: subgranular zone (SGZ), granule cell layer (GCL). (TIF) [file pone.0059250.s001.tif]

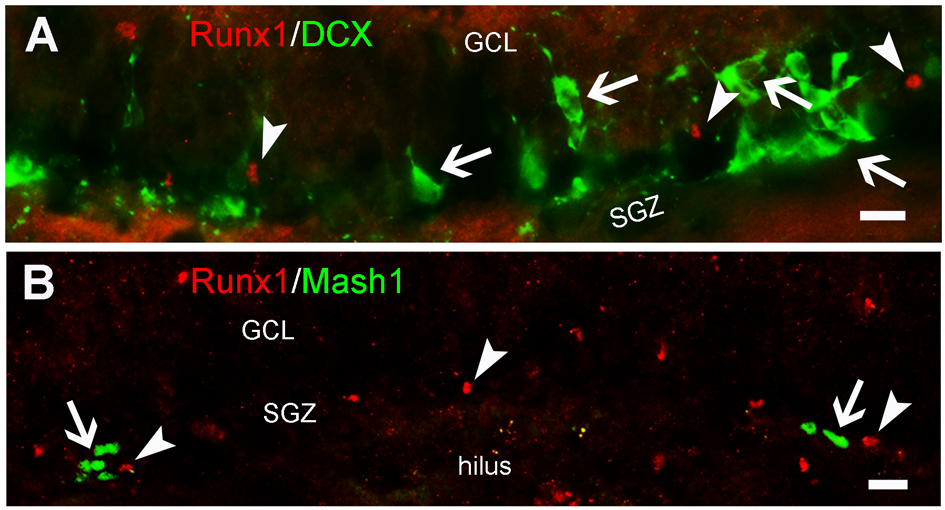

Supplement: Figure S2 — Runx1 is not expressed by DCX+ or Mash1+ cells of the DG. Merged images showing Runx1+ cells (arrowheads) and DCX+ (A) or Mash1+ (B) cells (arrows) in the dentate gyrus at 1 day post-injury (dpi). Scale bars = 20 µm. Abbreviations: granule cell layer (GCL), subgranular zone (SGZ). (TIF) [file pone.0059250.s002.tif]
